# Update on the pathogenesis of atopic dermatitis^☆^

**DOI:** 10.1016/j.abd.2024.06.001

**Published:** 2024-08-12

**Authors:** Paulo Ricardo Criado, Hélio Amante Miot, Roberto Bueno-Filho, Mayra Ianhez, Roberta Fachini Jardim Criado, Caio César Silva de Castro

**Affiliations:** a Centro Universitário Faculdade de Medicina do ABC, Santo André, SP, Brazil; b Faculdade de Ciências Médicas de Santos (Centro Universitário Lusíada), Santos, SP, Brazil; c Department of Dermatology, Faculdade de Medicina de Botucatu, Universidade do Estado de São Paulo, Botucatu, SP, Brazil; d Division of Dermatology, Department of Internal Medicine, Faculdade de Medicina de Ribeirão Preto, Universidade de São Paulo, Ribeirão Preto, SP, Brazil; e Department of Dermatology, Hospital de Doenças Tropicais de Goiás, Goiânia, GO, Brazil; f Alergoskin Alergia e Dermatologia, UCARE Center and ADCARE, Santo André, SP, Brazil; g Pontifícia Universidade Católica do Paraná, Curitiba, PR, Brazil; h Hospital de Dermatologia Sanitária do Paraná, Curitiba, PR, Brazil

**Keywords:** Atopic dermatitis, Cytokines, Dermatopathies, Eczema, Homeopathic pathogenesis, Immunity

## Abstract

Atopic dermatitis is a chronic, recurrent, and multifactorial skin-mucosal manifestation resulting from the interaction between elements mainly associated with the skin barrier deficit, the homeostasis of the immune response, neurological aspects, and patterns of reactivity to environmental antigens, which are established in genetically predisposed individuals. In addition to the skin, atopic diathesis involves other organs such as the airways (upper and lower), eyes, digestive tract, and neuropsychiatric aspects, which inflict additional morbidity on the dermatological patient. The different phenotypes of the disease fundamentally depend on the participation of each of these factors, in different life circumstances, such as age groups, occupational exposure patterns, physical activity, pollution, genetic load, and climatic factors. A better understanding of the complexity of its pathogenesis allows not only the understanding of therapeutic targets but also how to identify preponderant elements that mediate disease activity in each circumstance, for selecting the best treatment strategies and mitigation of triggering factors. This narrative review presents an update on the pathogenesis of atopic dermatitis, especially aimed at understanding the clinical manifestations, the main disease phenotypes and the context of available therapeutic strategies.

## Introduction

Atopic dermatitis (AD), or atopic eczema, is a chronic, recurrent, and multifactorial inflammatory disease characterized by eczema, dry skin (xerosis), and pruritus in variable degree, but generally intense, which compromises different dimensions of quality of life: symptomatic, functional, emotional, interpersonal and professional relationships. Due to its high prevalence, especially in childhood, in addition to quality of life, it impacts family dynamics and costs of the healthcare system.^1^, ^2^, ^3^, ^4^

The scientific knowledge about the different aspects of AD and atopic diathesis has advanced considerably in recent years. Its pathogenesis is complex, involving immune-mediated mechanisms, and its understanding is advancing in relation to genetic predisposition, structural and functional changes in the epidermal barrier, innate and adaptive immune responses, colonization of the skin by microorganisms, bacteria and fungi, reactivity to house dust mites, neurobehavioral elements, and triggers for exacerbation of the subclinical disease.^5^

Numerous genetic-based factors and environmental stimuli interact in the same individual, leading to the triggering or worsening of AD. Such complexity of elements justifies the variability of its phenotypic expression, intensity, and clinical course. Ethnic, geographic, climatic, pollution, and occupational factors comprise the main circumstances that contribute to this variability in the clinical presentation of AD. There is also variation in the clinical picture related to patients age group, which also correlates with the pattern of environmental exposure and other atopic conditions (allergic asthma and rhinitis, food allergy, allergic conjunctivitis, and eosinophilic esophagitis).^5^, ^6^

This article constitutes a multidisciplinary narrative review of the current aspects of AD pathogenesis, which, although not fully elucidated, can be understood based on the interaction of unique aspects such as genetic predisposition; changes in the skin barrier; activation of keratinocytes by mechanical, physical or chemical stressors; imbalance of the innate and adaptive immunity responses/loss of immune tolerance; intrinsic and extrinsic factors as disease triggers or aggravating factors; associated organic comorbidities, and sensitization of the pruritus neural pathways, so that a bidirectional neural and immune interaction is established in AD.^5^, ^7^

## Atopic diathesis

Hypersensitivity is the most common comorbidity of AD. About 70% of children with AD are sensitized to one or more antigens. Moreover, atopic diathesis has a strong genetic burden. Most children with AD have at least one parent with respiratory allergies, skin allergies, or both. The child of one allergic parent is two to three times more likely to develop atopy, which increases to three to five times more when both parents are allergic.^8^

The main route for sensitization and perpetuation of food-induced hypersensitivity lesions is the cutaneous one. The gastrointestinal route, mainly in the interval between the 4th and 6th months of life, is generally associated with the induction of tolerance, as regulatory T and B cells have been described as playing a role in regulating the pathogenesis of AD and food allergy, as well as in the development of tolerance to the foods involved.^9^, ^10^

The skin barrier dysfunction that occurs in AD increases permeability to allergens and irritants, contributing to loss of hydration and persistence of subclinical skin inflammation.^9^, ^11^ The formation of subclinical inflammatory lesions and the absorption of food antigens through the skin, whether due to contamination in dust or the use of products containing food proteins, leads to sensitization in infants with AD.^9^

Based on the findings of barrier alteration and the development of food allergy, the prophylactic use of emollients in children with AD and the risk of food allergy was idealized; however, the BEEP study showed that emollients can increase the risk of AD due to the colonization of pathogens on the skin, and *S. aureus* colonization is associated with an increased risk of food allergy.^11^

In a murine model, skin colonized by *S. aureus* leads to the activation of dermal antigen-presenting cells after exposure of inflamed skin to exogenous food allergens, expansion of germinal center IgE + B cells, and generation of IgE specific to food allergens, in addition to inflammatory cytokines (IL-4, IL-5, thymic stromal lymphopoietin (TSLP) and IL-33). Furthermore, it was demonstrated that epicutaneous exposure combined with staphylococcal enterotoxin B increased allergenic responses to eggs and peanuts. Thus, cutaneous microbial dysbiosis also contributes to barrier dysfunction, modulating allergic sensitization.^12^

Therefore, infants with eczema are exposed to food antigens through the skin, inducing immune cells, and producing IgE antibodies (allergic sensitization).^13^ This implies the importance of not only inducing oral immunological tolerance but also preventing allergic sensitization through the skin, aiming to reduce other hypersensitivities.^10^ Allergy to peanuts was also shown to be associated with the use of skin products containing peanut oil in England.^10^ It is important to highlight that, in Brazil, many oils used for skin hydration contain food antigens such as peanut, coconut, almond, and grape oil, among others.

In the HealthNut study, 5,276 children with AD were monitored during the first four years of life, demonstrating an increased risk of developing food allergies, such as peanut and egg allergies.^13^ A systematic review involving 66 studies revealed that food sensitization in children with AD was six times greater compared to healthy controls at three months of age.^10^

Several studies have confirmed an association between food allergies and more severe AD, as well as an association between the early and persistent development of AD and food allergies, observing a greater likelihood of sensitization to common foods such as milk, eggs, and peanuts. In short, early-onset AD is related to the development of food allergies. Conversely, food allergy worsens AD or increases the risk of comorbidities, including anaphylaxis.^8^, ^10^, ^14^

Upper and lower-airway allergies are common in patients with AD. *Dermatophagoides pteronyssinus*, *Dermatophagoides farinae*, pollen, and cockroaches are the most relevant allergens. However, as AD has different immune response profiles, cases without association with asthma and rhinitis are frequent, especially in late-onset ADs, with a more cellular response than an IgE-dependent one.^15^

Exposure to aeroallergens can trigger skin reactions in AD, suggesting direct contact with the skin as the main stimulus route. There are several possible mechanisms for aeroallergens to trigger AD. By penetrating the compromised skin barrier of AD, intact allergens can be captured, processed, and presented by antigen-presenting cells via IgE-facilitated presentation.^14^ The IgE-antigen complex is then internalized and degraded, with subsequent antigen presentation in the major histocompatibility complex class II to T lymphocytes.^14^ In fact, serum IgE values ​​are associated with respiratory, but not cutaneous, disease activity.^14^ However, allergens can also be phagocytosed and presented for recognition by T lymphocytes, respectively, without IgE involvement.^15^

AD is also associated with Ocular Surface Disease (OSD). Between 25% and 50% of patients with severe AD (Odds Ratio [OR = 2.17]), asthma and rhinitis (OR = 1.76), and childhood-onset AD (OR = 1.34) are associated with OSD. The immunopathogenic mechanism involves adaptive T-cell immunity against allergens or pathogens on the ocular surface. Clinically, OSD includes conjunctivitis, keratoconjunctivitis sicca, blepharitis, keratoconus, and infectious and non-infectious keratitis (corneal inflammation), with conjunctivitis being the most common among them.^16^

The most common conjunctivitis subtypes are allergic (seasonal and perennial), followed by papillary, vernal (chronic inflammation of the outer edge of the palpebral conjunctival mucosa), atopic blepharoconjunctivitis (due to inflammation of the eyelid and surrounding conjunctiva), atopic and infectious keratoconjunctivitis. Atopic keratoconjunctivitis is associated with a risk of progressive corneal damage and visual loss. There is a high risk of bacterial conjunctivitis (from *Staphylococcus spp*.) and viruses (adenovirus and *Herpes simplex*). Conjunctivitis in patients with AD requires a multidisciplinary approach due to the risk of visual impairment.^16^

Treatment with anti-IL-4/IL-13 biologicals is associated with conjunctival inflammation, especially after three months of dupilumab use, in a restricted number of adult patients (6%–30%), which occurs especially when high basal serum levels of Thymus and Activation-Regulated Chemokine / CCL17 (TARC) and total IgE are observed. These cases progress with a decrease in globose epithelial cells in the conjunctiva, with a reduction in mucin production, increased infiltration of immune cells, tear film instability, barrier dysfunction, and conjunctival inflammation.^16^

AD is usually the first step in the development of the so-called “atopic march”, as the epidermal barrier dysfunction favors the permeation of antigens associated with its onset. The atopic march refers to the sequential progression to allergic rhinitis, food allergy or asthma; however, even such progression has a genetic basis. In a study carried out in the USA, in 18,596 children with AD, it was observed that African-American children showed progression from AD to asthma, while those of European-American descent progressed from AD to allergic rhinitis and Asian children progressed from AD to IgE-mediated food allergy. The risk of asthma among children of African-American ancestry was six times higher than among children of European-American ancestry.^17^

The TSLP alarmin produced by keratinocytes subjected to stress, allergens, or pathogens, causes the activation of dendritic cells and the secretion of chemokines, which attract type 2 helper T lymphocytes (Th2) to the skin, releasing pro-allergenic cytokines (type 2 inflammation cytokines, such as IL-4, IL-13, IL-5, IL-31). In AD, the unregulated and excessive production of cytokines, especially IL-4 and IL-13, causes the inhibition of the expression of epidermal proteins such as filaggrin, loricrin, and involucrin, which reinforces defects in the epidermal barrier, favoring the permeation of allergens and pathogens, feeding back the disease. The dysregulated type 2 inflammation cytokine response occurs in different anatomical sites of the body. At the level of the bronchi, IL-4 and IL-13 act by mediating inflammation and remodeling changes in the airways, predisposing the development of type 2-related asthma (50%–70% of asthmatics).^18^

In the context of chronic rhinosinusitis (CRS), damage to the upper airway epithelial barrier initiates and perpetuates type 2 inflammation, and environmental factors such as viruses and other stimuli generate alarmins such as TSLP, IL-25, and IL-33, which increase nasal inflammation. Among asthmatic patients, there is a prevalence of approximately 40% of CRS with polyposis, and AD is more common among these patients with CRS with nasal polyposis, compared to those with CRS without polyposis. This type 2 inflammation mechanism is believed to be shared simultaneously, predisposing and amplifying the host immune response to external stimuli, as it occurs in allergic conjunctivitis and eosinophilic esophagitis.^18^

## Genetics of atopic dermatitis

AD is more prevalent in some families, with genetic factors accounting for 82% of the risk of developing AD, versus 18% for environmental factors.^19^ Moreover, children with both parents with a history of atopy are five times more likely to develop early-onset AD and persistent phenotype, compared to children with parents without AD history.^20^

The heritability of AD in monozygotic and dizygotic twins is 72%–86% and 21%–23%, respectively.^21^ The clinical subgroup most often associated with genetic transmission is patients with early-onset and resolution in adolescence, in addition to males.^22^

The high variation in prevalence and the different characteristics of atopic diathesis associated with ethnicities, such as the increased genetic heritability of asthma in black children, and the low prevalence of AD in Central Asian populations, have stimulated dozens of studies on AD and population groups.^23^, ^24^ In spite of this fact, larger genome-wide research is needed to rigorously explain how ancestral-based genetic alterations act at a genetic-social interface.^25^

A study with Mendelian randomization identified an association between age at onset of diabetes and also alcohol use with AD.^26^ A recent systematic review of 30 studies with Mendelian randomization determined that body mass index, intestinal flora, gastroesophageal reflux, and the IL-18 pathway were causal factors for AD, while AD was a causal factor for comorbidities such as heart failure, rheumatoid arthritis, and conjunctivitis.^27^

In Brazil, the general prevalence of AD in indigenous communities (1.9%) was lower than that observed in the population of big cities, despite not being stratified by age, and the fact that there is an expectation of a higher proportion of children in less urbanized areas.^2^, ^28^

### Association of candidate genes

There are dozens of AD susceptibility loci characterized in diverse populations, which mediate functions such as response to interferon gamma (IFNγ), innate immunity, T lymphocyte functions, and epidermal barrier dysfunctions (Table 1).^26^, ^29^, ^30^ Similarly, the most relevant associations of signaling pathways analyzed by the Reactome database were those of the interleukins IL4/IL-13, IL-2 and IL-12.^31^ It is estimated that up to 15% of AD heritability is determined by common variants and 12.5% ​​by rare variants at these loci.^32^

**Table 1:** Main mutations associated with atopic dermatitis, regardless of the studied population.^30^

Gene/Mutation	Odds ratio (95% CI)
FLG rs558269137/2282del4	3.75 (2.39–5.88)
FLG rs61816761/R501X	2.46 (1.48–4.10)
IL-18 rs187238/-137G/C	2.06 (1.37–3.10)
IFNγ rs2430561/T874A	1.93 (1.12–3.33)
TLR2 rs5743708/R753Q	1.82 (1.06–3.12)
Chr11q13.5 rs7927894	1.36 (1.11–1.67)
TLR2 A rs4696480/A-16934 T	1.26 (1.03–155)
SPINK5 rs2303063/Asn368Ser	1.26 (1.07–1.50)
IL-17A rs2275913/-G152A	1.14 (1.01–1.30)
IL-4 rs2243248/-1098T/G	0.47 (0.34 ‒ 0.65)

The filaggrin gene (FLG) shows a great diversity of ethnic-dependent genetic mutations and is the most frequently studied gene in AD studies, and certain variations are associated with more severe forms of the disease.^31^ In highly miscegenated populations, such as in Brazil, mutations associated with different ancestries can be identified.^33^

Furthermore, the FLG has mutations associated with the earlier onset of the disease (as does NLRP10), while other SNPs in genes such as *AFF1* and *EHMT1* increase susceptibility to the later onset of AD.^34^, ^35^ A meta-analysis of candidate gene studies in Europeans and Asians confirmed the association between *FLG* variants and AD risk and further revealed new associated loci in Europeans, *IL-18* and *TGFB1*, and in Asians, *IL12RB1* and *MIF*.^36^

Changes in the SPINK5 gene also lead to changes in the skin barrier, as they are related to the endogenous protease inhibitor LEKTI, which plays an important role in the homeostasis of the skin barrier, including epidermal desquamation and constitution of the lipid barrier. Several SPINK5 polymorphisms are associated with AD incidence in Chinese individuals.^37^

Regarding environmental factors, an association between AD and air pollution was identified in 19 genes linked to oxidative stress, which are mainly associated with the activation of neutrophils, and regulation of T helper lymphocytes and T regulatory lymphocytes (TREGs).^38^

Whole-genome studies have also identified genes associated with AD complication phenotypes, such as eczema herpeticum, for example *SIDT2* and *RBBP8NL*, which participate in the keratinocyte response to herpes virus type 1 infection.^39^ Also, paradoxical eczema in patients with psoriasis using immunobiologicals was associated with variants that can influence the expression of transcripts or the amino acid sequence in the IL-12 pathway, but not in the IL4/13 and IL17/23 pathways. Similarly, the authors speculate that IL-12 inhibition by genetic variants of this pathway enables the development of the Th2 response, which initiates paradoxical eczema.^40^

### Epigenetic factors

Epigenetics is the set of changes in gene expression that can be inherited or not, but do not alter the DNA sequence. Environmental stimuli (e.g., pollution, diet, smoking, radiation, chemical agents) are the main epigenetic regulators. The main epigenetic studies in AD are related to the DNA methylation of two genes, *NLRP2* (involved in macrophage activity) and PITPNM2 (related to neutrophils), both associated with smoking. Also, the association of eczema herpeticum and serum IgE levels with methylation in the IL-4 and IL-13 genes.^41^, ^42^, ^43^ Furthermore, there are microRNAs that are up- or downregulated in the skin or serum of patients with AD, such as MiR-144, associated with early onset of the disease.^31^

## Skin barrier

The skin constitutes the largest organ of the human being and it functions as a protective barrier separating the body from the external environment, protecting humans from thermal, actinic, osmotic, chemical, infectious, and mechanical aggressions. The epidermis, dermis, and subcutaneous tissue together with eccrine glands, pilosebaceous follicles, and other skin appendages form an integrated structure that interacts, promoting this protective function.

The multilayer structure, which is one of the main characteristics of the skin barrier, performs interdependent functions. The stratum corneum is the outermost portion of the skin, which acts as the main physical barrier, in an arrangement that is similar to bricks (corneocytes, with polar chemical characteristics) and cement (intercellular substance, predominantly lipids and ceramides, with nonpolar characteristics), which, together with the occlusion (tight junction - TJ) zones, consisting of claudins and occludins also expressed in the granulosa layer of the epidermis and in the intercellular space of the keratinocytes of the sweat gland ducts, regulate the inflow and efflux of fluids and electrolytes in and out of the skin.^44^

Cell-cell contacts, especially in the human epidermis, are essential for tissue structuring and homeostasis. Adherens junctions, consisting of cadherins (trans- and cis-cadherin dimers) and nectins, mediate cell-cell adhesion. The TJs, including their claudins, occludins, and junctional adhesion molecules (e.g., ZO-1), establish the apical-basolateral polarity of keratinocytes and regulate the paracellular (intercellular) transport of ions and solutes. The expression of subunit proteins such as claudins and occludins is reduced by exposure to allergens and staphylococcal strains, compromising barrier integrity in AD.^45^, ^46^

Thus, the epidermal structure of intercellular adhesion performs a complex protective function, which, together with the cutaneous immune system, contributes to the first line of defense against microbial pathogens and environmental insults.

A dysfunctional skin barrier is more prevalent in patients with AD (Table 2), and this deficit has several clinical consequences, as these patients are characterized by high reactivity to environmental stimuli and pathogens, reinforcing the role of restoring the skin barrier and protection against triggers for disease control. Transepidermal water loss results in skin dryness and desquamation, a high pH increases bacterial proliferation, while less adhesion between the corneocytes favors the penetration of allergens and irritants, which can trigger an exacerbated inflammatory response.^47^

**Table 2:** Skin barrier characteristics in atopic dermatitis.

Characteristic	Alteration
pH	Increased
Transepidermal water loss	Increased
Skin permeability	Increased
Intercellular cohesion	Reduced
Filaggrin	Reduction / Dysfunction
Ceramides/Lipids/Cholesterol	Reduction
Kallikrein activity	Increased
Intercellular junction proteins (claudins/ocludins)	Reduction
Microbiome	Lower diversity / *S. aureus*

Barrier functions in AD patients vary at different stages of the disease. Using pH and transepidermal water loss (TEWL) measurements, differences in these parameters were observed between eczematous skin and apparently healthy areas in AD patients. Notably, even unaffected skin in AD patients showed differences when compared to healthy subjects skin. In AD, there is an average increase in pH from 4.99 to 5.68 and TEWL increases from 6 to 30 g/m^2^h.^48^

One of the main genetic factors associated with AD is the mutation in the *FLG* gene, which produces function impairment leading to a compromised skin barrier. Filaggrin is a monomeric subunit of a protein packaged in keratohyaline granules, produced in terminal keratinocytes, involved in the maintenance of the skin permeability barrier.^49^ This structural protein binds to keratinocyte filaments to increase the density of filament bundles, flattening the keratinocytes to their terminal shape, crucial for skin strength and integrity.^49^ Profilaggrin, produced from the *FLG* gene, is broken down into monomeric filaggrin, which in turn is degraded by proteases into free amino acids, functioning as natural hydration factors to retain water, maintain the skin pH and strengthen the stratum corneum, offering chemical and microbial protection.^49^

Mutations in the *FLG* gene can result in functional loss or a smaller number of intra-exonic repeat units, which lead to protein deficiency, altering corneocyte morphology. This compromises the para-cellular barrier, allowing allergens and haptens to pass through. Although *FLG* mutations can contribute to AD, they are not sufficient to cause the disease alone, as some individuals with null alleles do not develop it.^50^

Around 25% to 50% of AD patients have a mutation in the *FLG* gene as a predisposing factor, especially in extrinsic phenotypes.^50^ However, it is known that this variation in the gene loss of function has ethnic variations, with values ​​reaching 50% in Europe and 25% in Asia; however, African-American patients have a lower frequency of the mutation, with the *FLG2* gene mutation being more impactful for the disease.^51^ *FLG* methylation is also correlated with AD risk, supporting the epigenetic contributions for disease development.^52^

The predominant Th2 imbalance in AD is implicated in reduced *FLG* expression, contributing to the barrier defect. Moreover, there is evidence that the expression of similar proteins, such as filaggrin-2 and hornerin, increase the risk of AD.^53^

Other molecules participate in the process of stratum corneum formation. With the loss of the nucleus, the cells are flattened and the keratin molecules become parallel, creating the cornified envelope connected with extracellular lipids. The cohesion strength of the stratum corneum depends on the formation of covalent bonds of lysine and glutamine, in which precursor proteins are incorporated into keratin, such as involucrin, cornifin, loricrin, keratolinin, and desmosomal proteins, such as envoplakin and periplakin.^54^

The lipid matrix of the stratum corneum is defective in AD skin due to an alteration in the expression of enzymes involved in the synthesis and processing of free fatty acids and ceramides. Lipid-rich lamellar granules in the granular layer of the epidermis provide the hydrophobic seal in the more superficial epidermal layers. The lipid matrix damaged after processing by lipid processing enzymes becomes a mixture of ceramides, cholesterol, and free fatty acids (FFAs). In cases of AD, there is a decreased amount of ceramides and those present are shorter.^55^ This reduction occurs in both affected and unaffected skin, especially in those with filaggrin abnormalities. Furthermore, a reduction in the relationship between ceramide and cholesterol was identified in the stratum corneum of these patients.^56^ This may be controlled by increased activity of lipid-processing enzymes or be a result of underlying Th2 inflammation, substantiating the importance of keeping subclinical inflammation under control to prevent recurrence, as demonstrated with proactive therapy.^57^, ^58^

The elevated pH of the stratum corneum and the increased activity of serine proteinase promote the inactivation and degradation of acid sphingomyelinase and β-glucocerebrosidase, which are the enzymes required for ceramide synthesis.^59^ Elevated serine proteinase activity reduces secretion from lamellar bodies through plasminogen activator type 2 signaling and results in the abnormal transfer of several substances that are secreted from the lamellar body.^60^ This is ultimately related to the reduction of extracellular lipids reported in AD patients.

In damaged skin, the lengths of the chains of ceramides, free fatty acids, and esterified fatty acids are also shortened, which results in abnormalities in the epidermal lipid organization and consequent changes in the permeability of the skin barrier. In patients with chronic AD, increased kallikrein activity can also lead to these changes in lipid structure by inducing degradation of very long-chain fatty acid elongation protein.^59^

In addition to genetic changes, chronic inflammation associated with AD favors skin barrier dysfunction, as discussed below. The release of cytokines such as IL-4, IL-13, and IL-31 contributes to changes in cell differentiation and proliferation in the epidermis. Likewise, the Th2 tone of the disease suppresses the production of antimicrobial peptides and IFNα, favoring bacterial and viral infections.

Skin barrier dysfunction fundamentally favors the impact of environmental elements on the pathogenesis of AD. The bidirectional interaction of the external environment with human health and disease is called exposome, complementing the lifelong interactions between genes and environment in the pathogenesis of diseases. It can be classified as (i) General external factors, such as climate, biodiversity, urbanization (household fungi and mites), and socioeconomic contexts; (ii) Specific external factors: allergens, pollutants (particulate molecules – PM10, nanoparticles, nitrogen dioxide, ozone), detergents, metals, pollens, tobacco smoke, diet, sweat, lifestyle factors and microbes, and (iii) ) Internal factors: inflammation, metabolism, and oxidative stress exposome.^61^

Exposomes are key regulators in the interaction between the dysfunctional epidermal barrier, microbiome, genome, and immune dysregulation in AD. This deregulation of the epidermal barrier initiates a vicious cycle of inflammation that becomes chronic in AD, which is supported by changes in the microbiome (dysbiosis) and its imbalance, demonstrating the importance of epidermal barrier homeostasis to prevent or restore skin health in AD.^61^

An example of environmental pressure that can determine alarmin secretion by keratinocytes in the epidermis is the exposure to high concentrations of particulate matter with an average size <2.5 µm emitted by forest fires, which disrupts the epidermal barrier by damaging structural proteins (filaggrin and E -cadherin), and lipids in the epidermis. In addition to particulate matter, acicular nanoparticles (diameter up to 270 nm) of titanium dioxide, but not globular nanoparticles of the same diameter, were internalized by normal human keratinocytes and produced pro-inflammatory cytokines, such as IL1-α, IL-1β, IL-6, TNF-α and IL-8, inducing skin inflammation.^62^, ^63^

Exposure to synthetic detergents is associated with an increased risk of eczema related to the working activities of cleaning professionals, potentially through TEWL. An increase in the number of cases of AD exacerbation has been observed due to the increased use of disinfectants during the COVID-19 pandemic.^61^, ^64^

Tobacco smoke, whether through active or passive exposure, delivers benzene to the skin, which interferes with the inhibitory ability of TREG cells to secrete IL-10 and exert anti-inflammatory effects, which results in a higher prevalence of AD in children with relatives who smoke.^61^

Moreover, allergens with proteolytic properties, such as certain pollens and group 1 house dust mites, promote disruption of the epidermal barrier and contribute to a vicious cycle of inflammation-microbial dysbiosis and skin barrier fragility.^61^, ^65^ These elements support desensitization therapies aimed at reducing reactivity to these agents, especially in the presence of respiratory allergies.

In general, the deficit in the skin barrier in AD is an important component of allergic sensitization in these patients, and also a relevant factor in disease exacerbations. In cases with controlled disease (i.e., with subclinical inflammation), exposure to irritants, dust, detergents, and hot baths/showers in winter are elements recognized as triggers of clinical recurrence, which reinforces the importance of maintaining therapies aimed at recovering the skin barrier and proactive strategies to prolong AD clinical remission.^57^, ^58^

## Immunopathology

### Histopathological changes

Although there are no histopathological findings pathognomonic of AD, which can discriminate it from other eczematous dermatitis, there are a series of changes that lead to its diagnosis in each form of the disease, within a clinical context.

Histopathological findings vary depending on the stage of the disease, presence of infection, treatment, and the severity of the skin lesions. However, some common histopathological characteristics are observed in many cases.

Acanthosis with spongiosis and hyperkeratosis are the most characteristic alterations in eczematous plaques. Acute, exudative forms may even contain intraepidermal vesicles, whereas chronic or prurigoid forms show minimal spongiosis, in contrast to marked acanthosis and hyperparakeratosis.

The dermal inflammatory infiltrate also varies with the type of eczema, is more intense in the acute form, and sparse in the chronic and prurigoid forms. In the upper dermis, typical lymphocytes (especially CD4) predominate in perivascular distribution, with sparse eosinophils. Epidermal exocytosis of lymphocytes, without the formation of microabscesses, is a frequent finding in AD histopathology.

Vasodilation and edema of the upper dermis are more common in acute eczematous lesions.

**Fig. 1:**
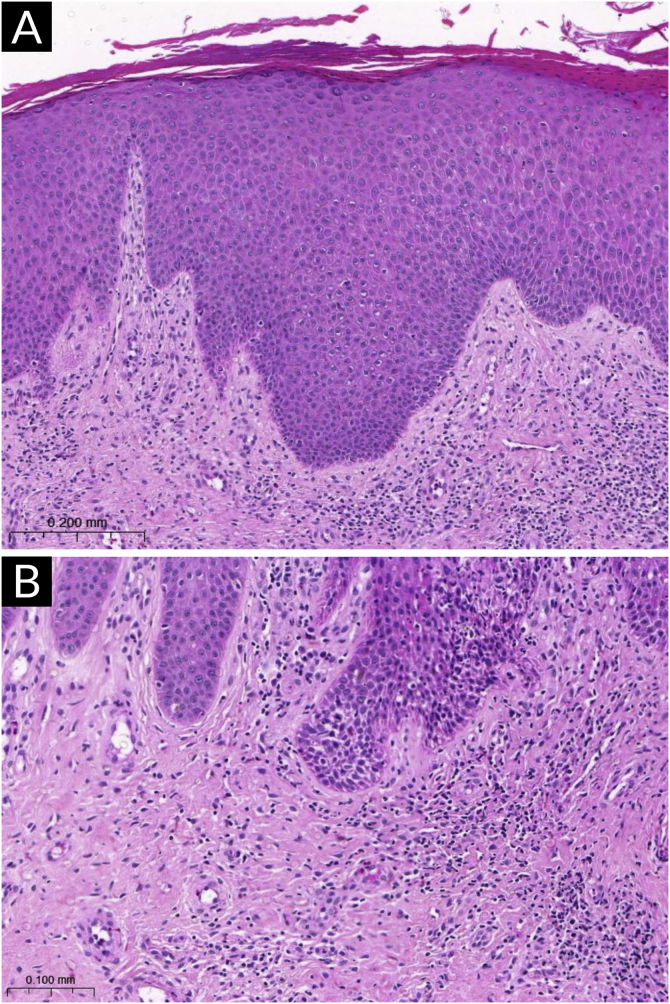
Histopathology of atopic dermatitis (subacute lesion). (A) Epidermis with irregular acanthosis, hyperkeratosis and focal parakeratosis, associated with mild spongiosis (Hematoxylin & eosin, ×200). (B) Inflammatory lymphocytic infiltrate of the superficial dermis, predominantly perivascular, with sparse eosinophils (Hematoxylin & eosin, ×400).

Fig. 1 exemplifies a subacute eczematous lesion of AD.

### Immune compartments of the skin

The skin is a secondary immune organ, and like the digestive tract and respiratory system, it represents a primary barrier of reactivity against environmental injuries and pathogens, which justifies its high reactivity to immunogenic stimuli. Non-specifically, it is believed that 95% of lesions are mitigated by the skin barrier, which reinforces the interaction of cutaneous immune system elements that contribute to AD pathogenesis.

Langerhans cells (LCs) are the main antigen presenters of the epidermis, immunocytes of the first line of defense, which phagocytose peptides through dendrites extended between keratinocytes. Also in the epidermis, resident memory T-cells provide a lasting immune response against known antigens, as they are located in the most likely sites for environmental injury and pathogens.

The dermis is the most active cutaneous immune microenvironment. In the papillary dermis, the loose connective tissue contains vessels and nerve endings, which branch between the keratinocytes. In the inflamed dermis, a diversity of immune cell types can be observed, such as various subtypes of T-cells, macrophages, dendritic cells, innate lymphoid cells, and mast cells, generally located around blood vessels and pilosebaceous follicles.

As the epidermal barrier is constantly exposed to environmental insults, the functions of the epidermis as an alarm sensor and integrator of environmental stimuli with the immune system are crucial. Cytokines derived from the epidermis, called alarmins, mediate a complex intercellular communication between epidermal keratinocytes and immune cells aiming at regulating immune surveillance.

### Alarmins

**Fig. 2:**
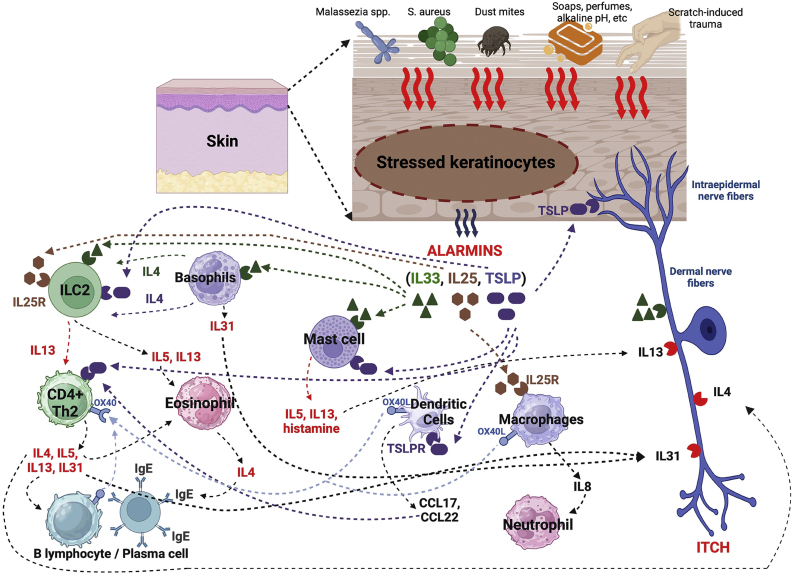
Production of alarmins resulting from stressors on keratinocytes and subsequent events in innate and adaptive immunity.

Alarmins are endogenous molecules that are part of DAMPs (damage-associated molecular patterns), which are important initiators of the inflammatory response. They function as damage signals (also known as stressors) and are released into the extracellular environment in response to epidermal damage aiming at triggering immune responses. Among them, there are alarmins with type 2 function, such as thymic stromal lymphopoietin (TSLP), IL-25, and IL-33, which are central orchestrators of immunity involving type 2 helper T lymphocytes (Th2). Its dysregulation or exaggerated and recurrent synthesis is associated with chronic inflammation, such as in AD (Fig. 2).^66^

A variety of endogenous and environmental factors, irritants, pollutants, and tobacco smoke stimulate keratinocytes to release TSLP. The activation of protease-activated receptor 2 (PAR2), toll-like receptors 4, and a member of the transient receptor potential channels sensitive to the valinoid (TRPV) family of channels, including TRPV1, on the keratinocyte cell membrane activates TSLP production through transcription factors, nuclear factor of activated T cells (NFAT), nuclear factor kappa B, and interferon regulatory factor 3.^66^

Various hematopoietic cells and sensory neurons express the TSLP Receptor (TSLPR). Furthermore, TSLP induces the expression of the major class I and II histocompatibility complex and in costimulatory molecules in dendritic cells, which produce Th2 cell-attractive chemokines such as TARC and CCL22 and induce Th2 differentiation through the enhanced expression of the costimulatory molecule OX40 L in the dendritic cell.^7^

OX40 and OX40 L are members of the tumor necrosis factor receptor /tumor necrosis factor (TNFR/TNF) superfamily. The members of the TNFR/TNF superfamily are immune checkpoint restriction molecules that can enhance T cell response through this co-stimulation. The OX40-OX40 L interaction allows a relevant co-stimulatory pathway in inducing the clonal expansion of effector CD4+ and CD8+ cells, intensifying the expression of pro-inflammatory cytokines, and facilitating the generation of memory T cells. The OX40-OX40 L interaction suppresses the production of TREGs, attenuating their suppressive function in inflammation/immune response. During the acute phase of inflammation in AD, OX40/OX40 L signaling promotes differentiation of Th2 cells, which express more OX40 and secrete cytokines that will promote rupture of the epidermal barrier, activating antigen-presenting cells that will subsequently express OX40 L. During the chronic phase of AD, other T cell subtypes express OX40 (Th1, Th17 and Th22), which are recruited to the inflammatory microenvironment. Effector memory T cells express OX40 and are overexpressed in AD lesions, which may offer a potential immune explanation for AD recurrence (exacerbations or flares) and AD chronicity.^67^

TSLP in keratinocytes can be stimulated by pathogens such as *S. aureus* and yeasts of the *Malassezia* genus, in addition to environmental stimuli, such as ultraviolet radiation, mechanical injury, and air pollutants. Moreover, vitamin D3 can induce the expression of TSLP in keratinocytes and develop an atopic dermatitis-like phenotype.^66^

IL-33 is predominantly produced by keratinocytes, which also express its ST2 receptor on their cell membrane. It is also produced by dermal fibroblasts and macrophages, expressed at high amounts in AD, where its serum levels are elevated compared to healthy controls and correlate with xerosis and excoriations.^66^

IL-33 is released by keratinocytes exposed to pathogens such as *S. aureus* and house dust mite allergens, in addition to environmental stimuli such as UVB and hypo-osmotic stress and mechanical epidermal damage, in addition to the induction of INFγ and TNFα, in keratinocytes. Nuclear IL-33 is elevated in TSLP-stimulated keratinocytes and IL-33 is required for the suppression of epidermal barrier integrity by TSLP, indicating that nuclear IL-33 is the key mediator in TSLP-induced epidermal barrier dysfunction.^66^

Keratinocyte-derived IL-25 is highly expressed in AD, and the production of IL-4, IL-13, IL-22, endothelin-1, and periostin increases IL-25 secretion by keratinocytes. Furthermore, IL-25 acts synergistically with other Th2 cytokines (IL-4 and IL-13) to suppress filaggrin expression in keratinocytes, exacerbating epidermal barrier defects in atopic dermatitis.

Alarmins also trigger the activation of inflammasomes in the skin. Inflammasomes are multiprotein complexes that form in the cytoplasm in response to danger signals and are part of the innate immunity in inflammation, removing pathogens and repairing damaged tissues. NLRP3 is the main inflammasome studied in AD; its formation is related to staphylococcal toxin and house dust mite allergens. The main cytokines directly induced by NLRP3 activation are IL-1β, IL-18, IL-5, and IL-31, which are associated with the Th2 response.^68^ In turn, inflammasome dysfunction can affect the skin barrier integrity, facilitating the penetration of allergens and microorganisms. Moreover, through its action on the nervous system, NLRP3 is also related to anxiety and depression behavior, frequently found in AD.^69^

### Immunopathogenesis

The pathogenesis of AD comprises the interaction between alterations in barrier function, and environmental stimuli in the context of a dysregulated and spectral Th2 and Th17/22 immune response.^61^

After surpassing the skin barrier, the antigens are phagocytosed by different antigen-presenting cells, especially LCs. During cutaneous inflammation, TNFα expression induces CXCL12 expression by dermal fibroblasts, and once LCs are in the dermis, they produce excessive amounts and express CCR7, enter the lymphatic system, and migrate to regional lymph nodes.^70^

This contribution of LCs also occurs in the context of AD and food allergy through transcutaneous sensitization to food allergens. With the capture of these allergens by LCs, presentation of the neoantigen to naïve CD4+ TLs in draining lymph nodes, and differentiation of CD4+ TLs into allergen-specific Th2 cells secreting pro-allergenic cytokines (IL-4, IL-5, IL-9, IL-13), together with type 2 innate lymphoid cells (ILC2s), act by modulating the activity of other dendritic cells, Th2 cells, eosinophils and mast cells.^71^

After the initial phase of immune response imbalance in AD, where alarmins, inflammasomes, epidermal and dermal antigen-presenting cells trigger type 2 inflammatory responses (innate responses with alarmins, ILC2s, and adaptive responses with cytokines IL-4, IL-5, IL -13, Il-31, in addition to the production of allergen-specific IgE), in chronic cases of AD, a dominance of Th1 and Th17 responses is gradually observed, mediated by the INFγ/TNFα axis and IL-17, respectively, while Th22 responses directed by IL-22 are also present.^61^

Thus, IL-4 and IL-13 constitute the main cytokines associated with the pathogenesis of early AD, as in addition to promoting type 2 inflammatory responses and recruiting eosinophils to the skin, they damage the epidermal barrier by suppressing the expression of structural proteins such as filaggrin, loricrin, and lipids, while concomitantly increasing collagen deposition in the dermis, which results in skin remodeling and lichenification.^61^

In addition to these actions, IL-4 and IL-13 contribute to (i) neurogenic pruritus by direct stimulation of these cytokine receptors in the pruritogenic sensory endings in the epidermis and dermis; (ii) stimulate the production of 3β-hydroxysteroid dehydrogenase-1 and androgens, which results in a decrease in the concentration of triglycerides in sebocytes and keratinocytes in the epidermis, contributing to damage in atopic skin, and (iii) make dermal mast cells secrete more IL-13 in response to skin damage and inhibit Th1-associated IL-12 production and subsequent release of INFγ, suppressing Th1-mediated responses to antigens found in AD.^61^

In addition to epidermal damage in AD and exposure to allergens activating Th2 immune responses, TREGs are deregulated, perpetuating the inflammatory response environment. This reduction and functional impairment of TREGs can be attributed to certain components of the exposome, such as tobacco smoke during pregnancy and dysbiosis of the skin microbiome. In AD, TREGs cells, after stimulation by staphylococcal superantigens (type B enterotoxin), lose immunosuppressive activity.^5^

Elevated serum levels of IL-21 are observed in patients with AD, primarily produced by Th17 cells, which is associated with greater AD severity when compared to healthy controls so IL-21 directly promotes type 2 inflammation and suppresses Th1 responses by suppressing INFγ production.^61^

IL-22, a Th22 cytokine, is elevated in both acute and chronic AD, damaging the epidermal barrier by suppressing proteins relevant to the normal differentiation of the epidermis, increasing sensitization to antigens and promoting an exacerbated type 2 immune response, in addition to contributing to the pathogenesis of pruritus, by promoting pruritus-inducing cytokines.^72^

The increase in the enzymatic activity of phosphodiesterase 4 observed in AD determines a reduction in intracellular cyclic adenosine monophosphate, which is a negative regulator of cytokine production, and is another element that contributes to the increased secretion of pro-inflammatory mediators involved in acute and chronic inflammation in AD.^61^

The aryl hydrocarbon receptor (AhR) is a transcription factor that acts in the regulation of multiple signaling pathways involving skin homeostasis, immune responses, and the epidermal barrier function. In the skin, AhR is expressed primarily in LCs and keratinocytes. In the absence of its activating ligands, such as certain polycyclic aromatic hydrocarbons, AhR is associated with a complex of plasma proteins, which include heat shock protein 90 and other chaperone proteins. AhR controls responses to xenobiotics (drugs) and environmental stressors, preserving homeostasis. This receptor is activated by a variety of low-molecular-weight ligands that can reach the epidermis endogenously, through diet, environment, and bacterial sources, such as arachidonic acid metabolites, indigoid compounds, heme metabolites, dietary factors such as flavonoids, carotenoids and metabolites produced by commensal intestinal bacteria. Polycyclic aromatic hydrocarbons (PAHs), dioxins, and polychlorinated biphenyls come from the environment. The activation of AhRs by different ligands can lead to the stimulation or suppression of different genes, causing a wide variety of biological responses.^73^

The physiological activation of AhR promotes the differentiation of keratinocytes and the formation of the epidermal barrier, with the synthesis of filaggrin, loricrin, and involucrin. Exposure to environmental pollutants, such as hydrocarbons present in tobacco, and solid or liquid particulate matter that remains in the atmosphere derived from the combustion of fossil derivatives (coal and oil), activates the AhR pathway, increasing the risk of developing AD. AhR activation in the human skin can cause excessive production of IL-22, which can potentiate AD activity. In AD, particulate matter originating from environmental contamination stimulates AhR to produce, in a harmful way, IL-33 (alarmin) in macrophages and keratinocytes, which tends to induce Th2 responses. Together with IL-4 and IL- 13, it suppresses the expression of filaggrin and loricrin, generating epidermal barrier dysfunction, and is a source of IL-33 for inducing pruritus by binding to ST2 receptors in epidermal and dermal sensory nerve endings. IL-33, in turn, has its production stimulated in keratinocytes via MAPK through positive feedback by cytokines produced by dermal inflammatory cells in AD, such as Th1 (via interferon-gamma production), Th17 (via IL-17) and Th2 (via IL-4 and IL-13). Moreover, IL-33 reduces the synthesis of IL-37 in the granulosa cell layer of the epidermis, which physiologically stimulates the production of the epidermal differentiation complex, including filaggrin and loricrin, further compromising the epidermal barrier. Coal tar, tapiranof, as well as certain flavonoids, reduce oxidative stress by suppressing pro-inflammatory cytokines and stimulating the restoration of the expression of epidermal barrier proteins.^73^

Innate and adaptive immunity and its cellular elements, such as basophils, eosinophils, and macrophages, significantly contribute to the pathogenesis of AD. Basophils participate in the initiation of AD through increased expression of IL-4 and its interactions with dermal keratinocytes and macrophages, resulting in epidermal hyperplasia and epidermal barrier dysfunction. Moreover, it was demonstrated that basophils can induce the secretion of IL-4 via allergen-dependent IgE, in addition to IL-31 independently of the stimulus of allergen-specific IgE (activated by TSLP), via macrophages that assume an M2 phenotype in AD. These M2 macrophages secrete IL-31 under TSLP stimulation and also periostin (secreted by fibroblasts), which could explain the residual pruritus in AD patients using dupilumab, a monoclonal antibody that blocks the IL-4Rα receptor and the IL-4Rα receptor coupled to IL-13 R.^72^

The number of M2 macrophages expressing IL-31, CD68+ macrophages, and basophils in biopsies of skin affected by AD is positively related to TSLP and periostin expression in the epidermis of affected skin in patients with AD and also to disease severity.^61^

Activated eosinophils in AD express high amounts of histamine type 4 receptors, through the stimulation of IL-4 and IL-13 via the JAK/STAT system, also leading to increased production of IL-31 by eosinophils. IL-18 acts on receptors (IL-18Rα) overexpressed on eosinophils in patients with AD and the histamine released by mast cells and basophils increases the expression of IL-18 on eosinophils by binding histamine with H_2_R and H_4_R, suggesting a relevant function of IL-18 and histamine in eosinophilic inflammation in AD.^61^

Mast cells produce several cytokines, such as IL-1,2,3,5,6,7,8,9,13,16,17 and IL-33. IL-33 directs type 2 inflammatory immune responses in AD. The secretion of IL-9 allows the survival of T cells and the cross-activation between mast cells and IL-13 contributes to type 2 inflammation, in addition to stimulating fibroblasts in collagen synthesis and the differentiation of B lymphocytes in the conversion of IgE synthesis.^61^, ^74^

NK cells participate in the secretion of different cytokines such as IL-9, IL-13, IL-21, IL-22, and IL-31. And IL-21 in particular promotes activation of B and T cells and their differentiation, as well as increased NK cell activity.^74^

Finally, the immunopathogenesis of AD is based on a deregulated immune response, in a multidimensional and interconnected way, affecting the response to antigens, the induction of inflammation, immune homeostasis disruption, disarray of the architectural integrity of the epidermis and dermis, producing a cardinal symptom, which is pruritus.^61^

**Fig. 3:**
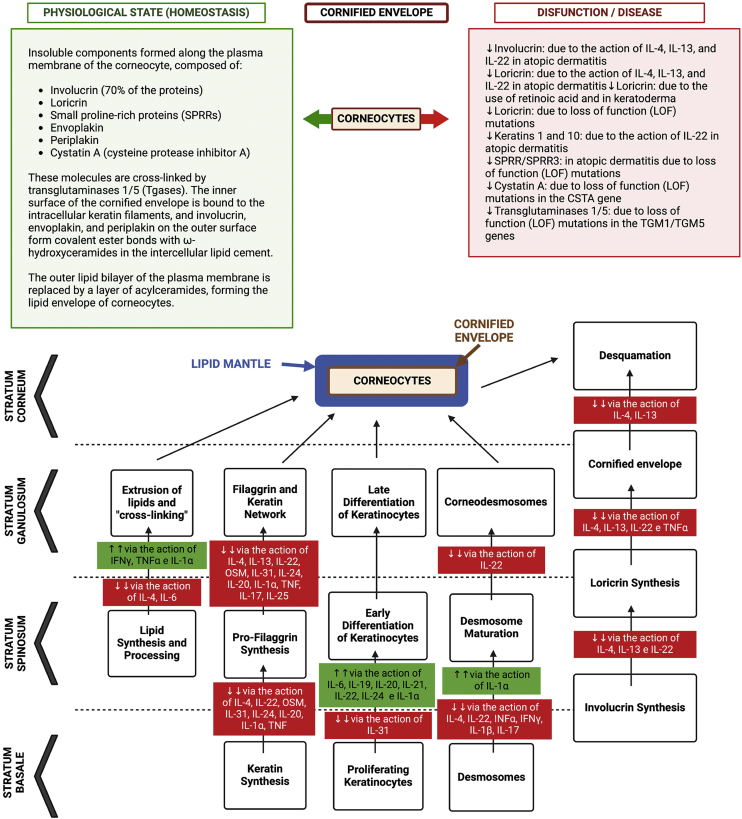
Representation of the differentiation process of keratinocytes in the epidermis, from the basal layer to the corneocyte, with epidermal barrier dysfunction due to the action of gene mutations with loss of function (LOF) and protein suppression due to the action of atopic inflammation cytokines. During this differentiation process, the lipid envelope, the filaggrin/keratin network, and the cornified envelope are formed, with the desmosomes differentiating into corneodesmosomes. Together these components form a compact barrier preventing the permeation of harmful substances or pathogens into the epidermis, as well as irradiation and irritants. Additionally, this barrier prevents transepidermal water loss (TEWL) and loss of associated solutes. The “bricks and mortar” model that occurs in the architecture of the corneal layer is comprised of the protein-lipid envelope that surrounds the corneocyte (proteins made up of loricrin, involucrin, small proteins rich in proline and filaggrin) and the lipid envelope made up of a monolayer of lipids, which function as the basis for the organization of intercellular lamellar lipids (consisting of 25% cholesterol, 10% to 15% free fatty acids, 5% of cholesterol sulfate and triacylglycerol and 45% to 50% of ceramides in the stratum corneum). These extracellular lipids are stored within lamellar bodies in keratinocytes of the upper spinous and granulosa layers, consisting of glucosylceramides, sphingomyelin and phospholipids. The “intercellular cement” is a matrix consisting of ω-hydroxyceramides, cholesterol and free fatty acids modified by enzymes from the stratum corneum, in addition to antimicrobial peptides. Free amino acids resulting from the degradation of filaggrin form the majority of the so-called “Natural moisturizing factor” (NMF) in the stratum corneum, representing an exceptional capacity to retain water and contributing to the acidic pH of this layer. Thus, the inter-corneocyte lipid-protein matrix and protein-rich corneocytes are crucial for the formation of the functional epidermal barrier. Genetic defects in genes that control the synthesis of these proteins can contribute to atopic dermatitis, as well as the suppression of the function of these genes by inflammatory cytokines, as observed in this diagram. The corneodesmosin (CDSN) gene is suppressed in atopic dermatitis, but the integrity of the corneodesmosomes can also be negatively modulated by type 2 cytokines: IL-4, IL-13, IL-31, IL-25, IL-22. IL-4 promotes filaggrin deficiency in AD. IL-33 suppresses claudin expression in keratinocytes. TSLP decreases the synthesis of antimicrobial peptides, such as human β-defensin via the JAK-STAT system and cathelicidin (LL-37), allowing greater vulnerability to eczema herpeticum and bacterial infections.

A summary of the interference of immune dysregulation regarding the skin barrier is depicted in Fig. 3.^75^

### Pruritus in atopic dermatitis

The cutaneous neurosensory system occupies a central position in AD pathogenesis. Sensory nerve endings are in intimate contact with resident and inflammatory cells that infiltrate the skin, so they can interact intensely with these cells and the mediators released in both the acute and chronic stages of the disease. Inflammatory mediators of AD can also sensitize sensory nerves, inducing the phenomenon of hyperkinesis, that is, determining an increase in the sensitivity of nerves to pruritogenic stimuli, and allokinesis (non-pruritogenic stimuli perceived as pruritus), contributing to chronic pruritus in AD.^76^

AD has the particularity of progressively developing an increase in the density of sensory neural fibers in the skin, causing a state of “neural sensitization”, resulting in greater reaction and interaction with the skin environment. Increased concentration of neurotrophins, released by nerve projections and skin eosinophils, act synergistically to amplify the branching of nerve endings in the skin, which results in hyperinnervation of inflamed skin in AD. This hyperinnervation may eventually decrease the pruritus induction threshold (hyperkinesis) and favor the induction of pruritus by non-pruritogenic stimuli (allokinesis).^76^

Unmyelinated, slow-conducting type C sensory neural fibers constitute the main pathways of cutaneous pruritus to the CNS. Basically, there are two pathways that conduct pruritus: histaminergic (histamine-sensitive) and non-histaminergic (histamine-insensitive) ones, anatomically inserted in type C fibers of the cutaneous sensory neuronal network. Antihistamine medications have been shown to have minimal or no effect on controlling pruritus in AD, provoking only somnolent effects on patients. This has been observed for decades, indicating that histamine plays only a minimal role in pruritus associated with AD, at least via stimulation of H1 receptors. Moreover, H4 receptor blockade has had a mild antipruritic effect in different experimental and clinical studies.^76^

Thus, pruritus in AD is primarily perceived by non-histaminergic pathways in the cutaneous sensory nerves, so inflammatory mediators that are pivotal in the pathogenesis of AD can stimulate non-histaminergic pathways and induce pruritus in AD. Irritants, allergens, and bacterial products generate proteolytic enzymes, which activate PAR2 receptors, by proteolytic cleavage of the extracellular N-terminal portion of this receptor. PAR2 receptors are located on keratinocytes and sensory nerves, so their stimulation is currently believed to be the main route for nonhistaminergic pruritus in AD and induction of neurogenic inflammation, which results in the release of neuropeptides such as substance P and calcitonin gene-releasing peptide (CGRP) through nerve endings in the skin (efferent neural flow), which feeds back to receptors in skin cells, intensifying inflammation.^76^

Exposure to several exogenous proteolytic enzymes (derived from bacteria or house dust mites) or from endogenous sources (tryptase, trypsin, chymase, kallikrein, especially KLK5) is observed in the skin of patients with AD, which are released by keratinocytes or by immune cells in the inflammatory process, highlighting the relevance of this pathway in the early stages of the disease. In the early phase of AD, PAR2 activation seems to precede the release of alarmins such as TSLP and TRPV3 stimulation can induce the release of TSLP. The latter, together with IL-33, are alarmins that stimulate type 2 inflammation and have receptors on the sensory neural endings of the skin, contributing to pruritus. Thus, keratinocytes transform exogenous and endogenous stimuli into pruritus signals via PAR2 and release mediators such as TSLP, creating the so-called “neuro-epidermal communication” in AD. The nerve endings, in turn, release substance P and calcitonin gene-related peptide (CGRP) in response. Substance P has receptors on the nerves themselves, on keratinocytes and inflammatory cells (lymphocytes, mast cells, eosinophils, and basophils), via high-affinity neurokinin receptor 1 and Mas-related G protein-coupled receptors (MrgprX2). The binding of substance P to MrgprX2 induces mast cell degranulation releasing pruritogenic agents such as leukotrienes, prostaglandins, histamine, TNF-α, proteases, and nerve growth factor (NGF).^76^

CGRP is a neuropeptide that stimulates sensory nerves, blood vessels and immune cells (dendritic cells and T lymphocytes), infiltrating inflammatory cells in the skin and propagating the Th2 immune response, releasing IL-13 from CLA + T cells in AD. Therefore, the efferent neural reflex (antidromic or opposite conduction of the stimulus) of neurogenic inflammation with local release of neuropeptides precedes the activation of the immune responses of the innate and adaptive systems, in relation to pruritus.^76^

When Th2 adaptive immunity is established, the synthesis of IL-4 and IL-13 plays a central role in type 2 inflammatory response in AD, with multiple effects on epidermal and dermal cells and sensory neural fibers. IL-4 and IL-13 sensitize pruritus-conducting sensory C fibers to lower the threshold of sensory stimulus perception to other pruritogenic stimuli, such as histamine, IL-31, and TSLP. IL-4 and IL-13, by suppressing the epidermal production of filaggrin, loricrin, and involucrin. This causes the release of proteolytic enzymes in the epidermis, that stimulate PAR-2 ​​receptors and release alarmins (IL25, TSLP, IL-33), as well as the selective expression of kallikrein (KLK0-7) in normal human keratinocytes, fueling inflammation and pruritus. These cytokines also amplify the immune response, as they have receptors in other lymphocytes, mast cells, basophils, and eosinophils, triggering the release of new or already pre-formed inflammatory mediators (histamine, tryptase, endothelin-1, eotaxin, IL-31) feeding chronic pruritus in AD. IL-31 has a special effect on the stimulation of pruritus in AD (pruritus cytokine), and has its receptor in the sensory nerves inducing pruritus, which in the alpha chain portion of the IL-31 receptor (IL-31Ra) is conjugated to the oncostatin M receptor beta subunit. Stimulation of both receptors (IL31Ra and OSMbeta) allows greater density and branching of sensory neural endings in the skin and increases sensitivity to stimulation by IL-31 and other pruritogens. The process of neural sensitization by IL-31 contributes to chronic pruritus in AD and has a critical role in the so-called “itch-scratch cycle”, a phenomenon that intensely promotes the development of prurigoid papular lesions, that is, nodular prurigo-type lesions.^72^, ^76^, ^77^

**Fig. 4:**
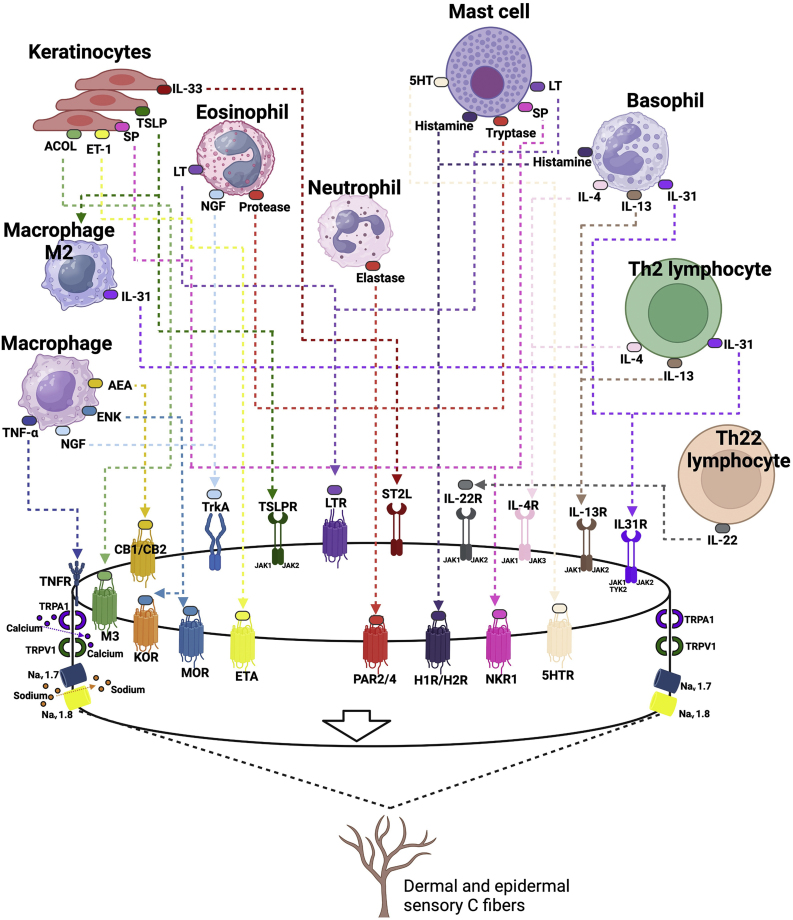
Pathways of pruritus, their mediators and receptors in atopic dermatitis. Prurigogenic agents produced by keratinocytes (substance P, SP; Acetylcholine, ACOL; endothelin-1, ET-1; and alarmins, such as thymic stromal lymphopoietin, TSLP and IL-33) bind to specific receptors in the membrane of slow conduction unmyelinated type C sensory nerves, respectively neurokinin-1 receptor, NK-1; muscarinic receptor, M3; endothelin 1 receptor, ETA (Endothelin A receptor); TSLP receptor, TSLPR; IL-33 STL2 receptor. The TSLP produced by keratinocytes under stress induces macrophages to differentiate into an M2 phenotype, which contributes to the production of interleukin 31 (IL-31). Undifferentiated macrophages produce tumor necrosis factor alpha (TNF-α), which binds to its receptor TNFR, anandamide (AEA) which binds to cannabinoid 1/2 (CB1/CB2) receptors, enkephalin, ENK, which binds to kappa (KOR) and mu (MOR) opioid receptors, in addition to producing nerve growth factor (NGF), which binds to the tropomyosin kinase A (TrkA) receptor. Eosinophils produce leukotrienes (LT), which bind to the LTR receptor, NGF and proteases that bind to the protease receptor (PAR2/PAR4). Mast cells release serotonin (5-hydroxy-tryptophan, 5-HT) activating its 5HTR receptor, histamine that binds to H1/H4 receptors, tryptase that binds to the PAR2/4 receptor, and substance P. Basophils are also a producing source of histamine and type 2 inflammation cytokines, such as IL-31, IL-4 and IL-13, which bind to their respective receptors in nerve endings, which have the JAK-STAT enzyme system as their intracellular signaling pathway. Th2 lymphocytes produce the same cytokines and Th22 lymphocytes produce IL-22, which also has its specific receptor on sensory nerve endings. All of them together work to produce acute and chronic pruritus in atopic dermatitis. The sensory neural fibers have transient potential receptors (TRP) V1 (valinoid transient potential receptor 1) and TRPA1 (ankyrin transient potential receptor 1), which are nonspecific cation channels. Once the nerve endings have been stimulated by the cytokines IL-4, IL-13, IL-22, IL-33, IL-31 and their specific receptors, the activation of TRVP1 and/or TRPA1 induces calcium influx, which eventually induces the release of action potentials via Na_v_1.7 and Na_v_1.8 or Na_v_1.9 sodium channels. TRPV1 and TRPA1 must be present for these pruritogens to induce pruritus or sensitize sensory nerves to other pruritogens.

A diagram of the pruritogenic stimuli in AD is shown in Fig. 4.

In the clinical disease presentation, these elements manifest themselves in their morphology as acute AD lesions comprising erythema, edema, and exudation, and chronic lesions as xerosis, lichenification, and hyperpigmentation.^74^

Initially, the early phase of AD was attributed to a Th2-driven response, while the chronic phase was marked by the predominance of Th1 responses.^74^ Moreover, acute lesions in AD reveal abundant lymphocytic infiltrate in the skin, as well as marked expression of IL-4, IL-5, IL-13, IL-31, and IL-33, which are characteristic of Th2 responses.^61^, ^74^

Nonetheless, subsequent studies showed that the Th2 response is simultaneously accompanied by Th22 activation and a smaller induction of Th17 response activation markers.^74^ Thus, the current understanding of chronic cutaneous forms of AD lesions demonstrates a simultaneous and feedback increase in the Th2 and Th22 cytokine axes, and, additionally, an increase in the expression of Th1 responses, but not specific markers of the Th17 response.^74^

These characteristics may be relevant in developing a therapeutic strategy and understanding the underlying immune response imbalance profiles. For example, considering ethnicity and AD clinical presentation, the role of the Th17 axis plays a greater role in the chronic phase of AD, and to a greater extent in intrinsic AD forms, in children and in patients of Asian ethnicity, where JAK1 inhibitors and/or anti-IL17, anti-IL-23, anti-IL-22 immunobiologicals may in the future prove to be an alternative to first-line therapeutic agents or in refractory cases. Likewise, phase III studies conducted with monoclonal antibodies targeting interactions with OX40L-OX40 co-stimulatory molecules in interactions between antigen-presenting cells and T lymphocytes can play a relevant role when a therapeutic action involving several active immune axes is intended, or even in populations with a high rate of racial miscegenation.^78^

**Fig. 5:**
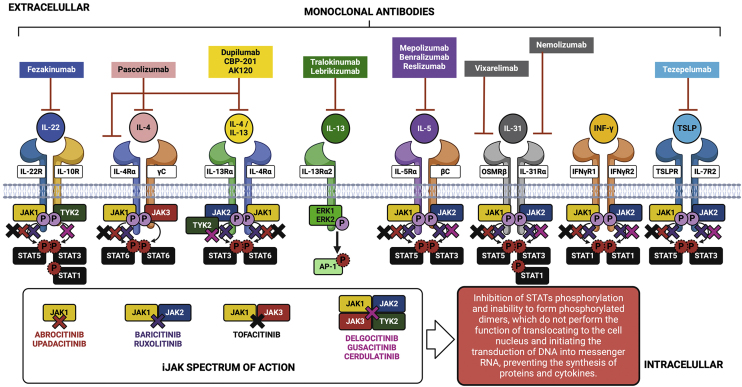
Main cytokines involved in the pathogenesis of atopic dermatitis, intracellular signaling of the JAK-STAT system, biologicals and small molecules used in the treatment of atopic dermatitis.

Knowledge about the action of cytokines through their specific cell receptors and the existence of the JAK-STAT intracellular signaling system in AD has led to the emergence of therapeutic strategies aimed at blocking cytokines or their extracellular receptors with monoclonal antibodies for subcutaneous use, and also the approach of partial interruption of the JAK-STAT system using drugs called small molecules for oral use, as shown in Fig. 5.

## Pathogenic basis of atopic dermatitis phenotypes

The polymorphism of the clinical presentations of AD is supported by a genetic basis, levels of skin barrier impairment, and patterns of immune response imbalance. Table 3, Table 4 summarize the different phenotypic aspects of AD and their relationship with serum IgE levels, in addition to patterns of more active immune responses in different ethnic groups. The recognition of these phenotypic aspects and their underlying pathophysiological basis can also guide the best therapeutic option.

**Table 3:** Main characteristics of AD phenotypes associated with IgE levels.

Characteristics	Extrinsic	Intrinsic
Serum IgE	↑↑↑	Normal
Frequency	70%‒80%	20%‒30%
AD onset	Childhood > Adulthood	Adulthood > Childhood
Filaggrin mutation	↑↑↑	↑
Ichthyosis vulgaris	↑↑↑	↑
Palmar hyperlinearity	↑↑↑	↑
Hypersensitivity	Asthma / Rhinitis, Food Allergy	Metal contact dermatitis
Skin infections	↑↑↑	↑
Preponderant response	Th2: IL-4, IL-13, IL-31	Th22: IL-22; Th17: IL-17
Severity markers	Th2 cytokines (IL-4)	Th17 cytokines (IL-17)
Dermal eosinophilia	↑↑↑	↑
Clinical predominance	Subacute eczema	Lichenification / Prurigo

**Table 4:** Main characteristics associated with the ethnicity of patients with atopic dermatitis.

Ethnicity	African/Black	Asian (Japanese, Chinese/Han, Korean)	European
Main genetic changes	FLG-2 (LOF), TCHH, TCHHL1, CLDN1, HRNR, CRNN, IL4, IL4RA, IL7R, IRF2, TSLP	FLG null, FCER1A, DEFB1, IL-4, IL-13, IL-31, IL-5, IL-12B, IL-18, IL-18RAP, IL-13RA1 TLR2, SPINK5	FLG (LOF), FCER1A, DEFB1, IL7R, IL-4, IL-13, IL-31, IL-4RA, IL-13RA1 TLR2, IRF2, TSLPR
Immune polarization	Th2 ↑↑↑, Th22↑↑, Th17 (+/−), Th1 (+/−)	Th22↑↑↑↑, Th2 ↑↑↑, Th17↑↑, Th1↑	Th2 ↑↑↑, Th22↑↑↑, Th1↑↑, Th17↑
Clinical predominance	Lichenification	Circumscribed lesions + lichenification	Extensive + malar eczema (childhood)
Therapeutic implication	Anti-Th2/Th22 strategies	Broad action strategy (immunosuppressants and JAK inhibitors)	Anti-Th2 / Th22 strategies

FLG, Filaggrin; ILC2, Innate Immunity Type 2 Lymphoid Cell; LOF, Loss-of-Function; IL, Interleukin; INF, Interferon; IRF2, Interferon Regulatory Factor 2; TNF, Tumor Necrosis Factor; TLR2, Toll-Like Receptor 2; TSLP, Thymic Stromal Lymphopoietin; FCER1A, IgE high-affinity receptor alpha subunit; DEF1, Beta-Defensin-1; SPINK5, serine peptidase inhibitor Kazal type 5; HRNR, Hornerin; CLDN1, Claudin 1; Th, T-helper lymphocyte; JAK, Janus Kinase; CRNN, Cornulin.

About 70% to 80% of adults with AD have elevated serum levels of IgE and specific IgEs to several allergens (extrinsic, allergic or exogenous phenotype), in whom the severity of skin lesions correlates with serum IgE levels. Moreover, clinical improvement in eczematous lesions has been reported in allergen-free environments with specific IgE. In spite of that, omalizumab is not effective in the treatment of extrinsic AD eczema, and AD should not be justified solely by the occurrence of elevated serum IgE. However, the presence of IgEs specific to certain allergens plays a role in the development of eczematous dermatitis resulting from IgE-mediated delayed-type hypersensitivity, functioning primarily as an amplifier of other factors involved in the pathogenesis of AD.^79^, ^80^

On the other hand, in intrinsic AD (endogenous, atopic, or non-IgE-allergic), no sensitization to environmental allergens is detected and serum IgE levels are normal. Instead, skin inflammation is often attributed to factors such as a compromised skin barrier, imbalances in the skin microbiota, and immune system dysfunction. Intrinsic AD tends to be more persistent and less responsive to conventional treatments. This phenotype may not meet the usual diagnostic criteria for AD (e.g.; Hanifin and Rajka), leading to diagnostic delay and inappropriate therapies.^81^

Some patients may also experience an intense and chronic pruritus sensation, known as neuropathic pruritus. This phenotype is associated with abnormalities in the peripheral and central nervous pathways, and the lower pruritus threshold and recurrent scratching may contribute to the inflammation persistence in AD.

It is important to note that intrinsic and extrinsic phenotypes are not mutually exclusive, and some patients may exhibit characteristics of both phenotypes or migrate from the predominance of one to the other, during the course of the disease. Recognizing these differences can be useful in guiding therapeutic management, especially regarding the identification of environmental triggers.

Morphologically, according to the skin lesion pattern, a network of different cytokines takes the lead in the inflammatory response, with therapeutic implications. In acute eczema, the lesions are supported by tissue expression of IL-1alpha and IL-1beta, IL-33, IL-4, IL-13, and IL-22. In subacute eczema, the main cytokines are IL-4, IL-13, IL-25, IL-31, IL-33 and TLSP. In chronic eczema, there is a predominance of Th1 and Th17 cytokines: IFNγ, TNFα, IL-17A, IL-17F.^82^

Finally, series from different original ethnic groups also share phenotypes linked to the protagonism of the pathogenic components of AD (Table 4). However, globalization and population miscegenation, as it occurs in Brazil, tend to reduce the preponderance of phenotypes linked to ethnicity in the clinical presentation of AD.^83^

## Microbiome in atopic dermatitis

Human skin is a rich ecosystem inhabited by a diversity of microorganisms, including bacteria, fungi, and viruses, with bacteria from the *Actinobacteria*, *Firmicutes,* and *Proteobacteria* phyla being the most prevalent ones. Fungi, such as *Malassezia spp*., and *Demodex spp*. mites are also important components of the skin microbiota. These microorganisms, mainly commensals, interact directly with the host, act as protection against pathogens, stimulate the skin barrier, and regulate the immune response; however, their imbalance can contribute to the exacerbation of AD.^84^, ^85^, ^86^

The composition of the skin microbiota starts at birth, differing between newborns born via the vaginal route and via cesarean section. This composition is influenced by several factors throughout life, including changes in skin pH, humidity, pollution, and sebum production.^87^, ^88^, ^89^ Changes in the microbiome may precede the development of AD, with puberty marking another phase of significant change due to the influence of sex hormones.^90^, ^91^, ^92^, ^93^

The skin microbiota participates in the maintenance of the skin barrier function, influencing cell differentiation and regeneration. For instance, coagulase-negative staphylococci, such as *S. hominis*, produce substances that inhibit the growth of *S. aureus*.^94^, ^95^, ^96^

Patients with AD frequently exhibit dysbiosis, manifested by a decrease in microbial diversity, with a skin environment that favors the growth of *S. aureus*, associated to an increase in skin pH and antimicrobial component deficiencies.^97^, ^98^, ^99^, ^100^, ^101^, ^102^

A healthy skin flora is necessary to maintain the microbial barrier, as certain bacteria in healthy skin suppress virulent bacteria and induce the expression of antimicrobial peptides (AMPs), controlling inflammation. The skin pH helps to determine skin microbial populations, and an increased pH can increase susceptibility to infections. A healthy flora, along with normal expression of skin proteins, enzymes, proteases, and balance of inflammatory cytokines, are crucial for maintaining a normal acidic pH of the skin.^99^

*S. aureus* infection inhibits the expression of late differentiation factors in keratinocytes, such as filaggrin, loricrin, keratin 1 and 10 and desmocollin 1, which can further compromise the skin barrier. Additionally, bacterial components of *S. aureus* are associated with altered intercellular lipid composition.^103^, ^104^

The treatment of skin diseases with antibiotics and antibacterial agents can be effective; however, prolonged use increases the risk of promoting bacterial resistance. Recent therapies, such as dupilumab and tralokinumab, favor skin microbiota rebalancing in patients with AD.^94^, ^105^ The use of probiotics, prebiotics, and postbiotics is also explored to fight skin dysbiosis.^95^

There is less information regarding intestinal microbiota and AD. The human intestine hosts more than 100 trillion microorganisms and is influenced by factors such as genetics, diet and medical treatments. The composition of the intestinal microbiota affects immune development and disorders can lead to conditions such as inflammatory diseases and obesity. Dysbiosis in the intestinal microbiota is linked to AD, characterized by a decrease in microbial diversity and an increase in the presence of harmful bacteria such as *Clostridium difficile*. This imbalance can lead to increased intestinal permeability, with dysfunction of the interepithelial occlusion zones in the intestine, allowing bacterial toxins to permeate the epithelium and impact the local and systemic immune response, including in the skin.^106^ Dietary factors, particularly the Western diet, contribute to intestinal dysbiosis and AD by altering the intestinal microbial genes and inflammatory responses.^107^

## Neuropsychiatric aspects

AD impacts the quality of life of patients and their families, whose chronic contingency manifests itself, neuropsychologically, in several ways, including anxiety, depression, disruptive behavior disorders, neurological development disorders, attention deficit/hyperactivity disorder (ADHD), autism spectrum disorder, and suicidal ideation, with these associations especially striking in severe cases of AD.^108^, ^109^, ^110^

The prevalence of ADHD in children with AD was estimated at 7.1% (95% CI 5.4–8.9%), while in children without AD, it was 4.1%, in an Israeli case series.^111^ These children experience sleep problems at higher rates than those with AD alone. Sleep deprivation in the first three years of life is linked to hyperactivity and lower cognitive performance at age six, demonstrating that insufficient sleep can have lasting effects on neurocognitive development.^112^ Mothers of infants with AD report more pronounced feelings of depression and anxiety compared to mothers of children without AD. Moreover, parents of children with AD report that disrupted sleep is directly related to their own levels of anxiety and depression.^113^, ^114^

A longitudinal study that followed 1,578 children until age 10 showed that AD in childhood and sleep problems are significant predictors of conduct and emotional disorders at age 10.^115^ Research also associates ADHD with the use of antihistamines (OR = 1.88; 95% CI 1.04‒3.39), although studies on cetirizine did not find any significant differences compared to placebo in the children’s behavioral and developmental assessments.^116^, ^117^

Moreover, the presence and intensity of pruritus are positively correlated with symptoms of depression, anxiety, and stress throughout life. The high incidence of mental health symptoms in children with AD may be partially due to the coexistence of other conditions. Studies have shown that atopic comorbidities are associated with higher prevalence rates of several psychological conditions, such as autism and ADHD.^110^

Adolescents with AD report high rates of shame, avoidance of social activities, and fewer friends, while children with AD are bullied and have more negative experiences with peers and teachers.^118^, ^119^ Mild to moderate AD is associated with a 29% to 84% increase in the risk of internalizing behavior problems from ages four to 16, although it is not directly linked to an increase in the risk of depression from ages nine to 18.^120^ Additionally, such behaviors in childhood are related to depression and anxiety in adulthood.^121^

A systematic review indicated that approximately one in six individuals with AD has clinical depression, one in four has depressive symptoms, and one in eight has suicidal ideation.^122^ Patients with AD have greater chances of clinical depression and depressive symptoms when compared to healthy individuals, albeit similar to people with other chronic dermatological disorders. Finally, almost one-third of parents of children with AD are depressed, and there is a high prevalence of suicidal ideation among patients with AD.^122^

Pharmacological treatment of AD reduces anxiety and depression scores in patients with moderate to severe disease, while non-pharmacological interventions lead to reductions in anxiety scores.^123^ Moreover, psychological interventions led to improved quality of life and reduced eczema and pruritus severity scores.^124^

An association was identified between AD and other neuropsychiatric disorders, including autism (OR = 2.14; 95% CI 1.39‒3.29), ADHD (OR = 1.78; 95% CI 1.21‒2.62), personality and behavioral disorders and schizophrenia (OR = 1.45; 95% CI 1.23‒1.72),^125^ in addition to a new association between AD and an increased risk of developing dementia (OR = 2.02; 95% CI 1.24‒3.29).^126^ This highlights the complexity and severity of the impact of AD on the mental health and quality of life of patients and their families.

Although the relationship between AD and neuropsychiatric conditions is highlighted, the underlying cause of these associations is not fully understood, as well as the interaction between dermatological symptoms (e.g., pruritus) and the worsening of neuropsychiatric symptoms. A better understanding of these dynamics should lead to primary and secondary prevention strategies to reduce the neuropsychiatric impact of AD, such as early interventions in high-risk patients and measures to improve the quality of life of patients and their families.

## Comorbidities in adults and elderly people with AD

The chronic inflammatory state can lead to major cardiovascular events (angina, acute myocardial infarction, cardiac arrhythmias, etc.), heart failure, venous thromboembolism, and lymphoproliferative malignancies in patients with AD.^27^

In a cohort study in the United Kingdom population, which evaluated 625,083 adults with AD followed for at least five years, without receiving immunosuppressive therapy, compared to another 2,678,888 adults without AD, matched by gender and age, it was observed that among adults with severe AD (10,543 patients) there was a 1.86-fold increased risk of non-Hodgkin's lymphoma and a 21.05-fold increased risk of cutaneous T-cell lymphoma.^127^

Patient care data in the United Kingdom between 1994 and 2025 demonstrated that adults with severe AD are at increased risk of myocardial infarction (Hazard Ratio/ Instantaneous Hazard Ratio [HR = 1.27; 95% CI 1.15‒1.39]), cerebrovascular accidents (HR = 1.21; 95% CI 1.13‒1.30) and pulmonary embolism (HR = 1.39; 95% CI 1.21‒1.60), when compared to adults without AD.^128^

Despite the known association between systemic comorbidities and AD, and the importance of identifying these conditions, the direct causal relationship between chronic inflammation in AD and such conditions is not yet known, nor is it known whether effective AD treatment reduces the incidence of these comorbidities.

## Final considerations

AD is a complex, chronic, and recurrent multifactorial condition that has variable phenotypes, resulting from the interaction between the genetic basis, skin barrier defect, immune response profiles, and reactivity to environmental exposure. A better understanding of its pathogenesis leads to the possibility of intervention in the preponderant worsening factors, in each circumstance, as well as to promoting the reduction of the basal disease activity, in subclinical cases, prolonging periods of remission.

## Financial support

None declared.

## Authors' contributions

Paulo Ricardo Criado: Study planning, drafting and editing of the manuscript, approval of the final version of the manuscript.

Roberta Fachini Jardim Criado: Study planning, drafting and editing of the manuscript, approval of the final version of the manuscript.

Mayra Ianhez: Study planning, drafting and editing of the manuscript, approval of the final version of the manuscript.

Hélio Amante Miot: Study planning, drafting and editing of the manuscript, approval of the final version of the manuscript.

Caio César Silva de Castro: Study planning, drafting and editing of the manuscript, approval of the final version of the manuscript.

Roberto Bueno-Filho: Study planning, drafting and editing of the manuscript, approval of the final version of the manuscript.

## Conflicts of interest

Dr. Paulo Criado: Advisory board - Pfizer, Galderma, Takeda, Hypera, Novartis, Sanofi; clinical research - Pfizer, Novartis, Sanofi, Amgen and Lilly; Speaker: Pfizer, Abbvie, Sanofi-Genzyme, Hypera, Takeda, Novartis.

Dr. Roberta Fachini Jardim Criado: Advisory board - Pfizer, Takeda, Hypera, Novartis, Sanofi; clinical research - Pfizer, Novartis, Sanofi and Lilly; Speaker: Pfizer, Abbvie, Sanofi-Genzyme, Hypera, Takeda, Novartis.

Dr. Mayra Ianhez: Advisory Board - Galderma, Sanofi, Pfizer, Novartis, Abbvie, Janssen, UCB-Biopharma, Boehringer-Ingelheim; Speaker - Galderma, Sanofi, Pfizer, Theraskin, Novartis, Abbvie, Janssen, Leopharma, FQM.

Dr. Caio Castro: Advisory Board - Sanofi, Aché, Sun-Pharma, Galderma. Speaker - Abbvie, Jansen, Novartis, Sanofi, Leo-Pharma. Clinical Research - Abbvie, Pfizer, Jansen, Sanofi.

Dr. Roberto Bueno-Filho: Advisory board - Janssen, Novartis; clinical research – Sanofi, Janssen and Lilly; Speaker: Pfizer, Janssen, Sanofi, Novartis.

Dr. Hélio Miot: Advisory Board – Johnson & Johnson, L’Oréal, Theraskin, Sanofi and Pfizer; clinical research - Abbvie, Pierre-Fabre, Galderma and Merz.
